# Ten Years of Community Treatment Orders in Western Switzerland: an Update

**DOI:** 10.1007/s10597-025-01486-5

**Published:** 2025-07-18

**Authors:** Stéphane Morandi, Charles Bonsack, Karim Boubaker, Benedetta Silva

**Affiliations:** 1 Community Psychiatry Service, Department of Psychiatry, Lausanne University Hospital and University of Lausanne, Lausanne, Switzerland; 2 Cantonal Medical Office, Directorate General for Health of Canton of Vaud, Department of Health and Social Action (DSAS), Avenue Des Casernes 2, 1014 Lausanne, Switzerland

**Keywords:** Coercion, Community treatment order, Involuntary outpatient treatment, Outpatient commitment, Epidemiology, Bayesian model

## Abstract

Community treatment orders (CTOs) are legal procedures that authorise compulsory community mental health care for people with severe mental disorders. Since their introduction in Switzerland in 2013, the Swiss Canton of Vaud (846′300 inhabitants) has been engaged in the monitoring of CTOs. This study describes the use of this measure in the Canton of Vaud over the 10 years since its introduction and examines the profile of people undergoing CTOs during this period, the form that these measures took and the factors associated with their duration and outcome. Between 2013 and 2022, 530 CTOs have been ordered. As their incidence remained low and fluctuated between 5 and 10 CTOs per 100′000 inhabitants, their prevalence rose from 5 to 25 per 100′000 inhabitants. The profile of people placed under CTOs was comparable to that observed in other studies. The measures concerned mainly Swiss male in their forties, single or divorced/separated, living independently and under legal guardianship. The main diagnosis was schizophrenia, schizotypal or delusional disorders. On the 31st of December 2022, 329 CTOs (62.1%) had been discharged. Their average duration was slightly more than two years. Among the 329 CTOs discharged at the 31st of December 2022, five groups were identified according to the reasons for discharge: CTO success (38.7%), breached conditions with (24.0%) and without involuntary hospitalisation (11.5%), death of the patient (13.0%) and other reasons for discharge (12.8%). Prototypical situations for each group were identified.

**Supplementary Information:**

The online version contains supplementary material available at 10.1007/s10597-025-01486-5.

## Introduction

Community Treatment Orders (CTOs) are legal procedures that authorise the compulsory provision of community mental health care to people affected by severe mental disorders. Even though their usefulness in promoting adherence to care and improving the state of health and functioning of people who have to undergo them is still hotly debated (Cossu et al., 2024), CTOs use is spreading worldwide (Mikellides et al., 2019). Introduced for the first time during the 60’s in the United States, CTOs are nowadays regulated in 75 jurisdictions, with important variations in terms of rates of use, legal criteria (“treatment criterion” versus “danger criterion”) and duration (Mikellides et al., 2019; Silva et al., 2019).

In 2013, CTOs were introduced for the first time in the Swiss legal framework (Article 437 of the Swiss Civil Code; The Federal Authorities of the Swiss Confederation 2024). Federal law gives the 26 Swiss cantons the option of providing CTO within their own health legislation and the responsibility for defining its content and limits. In the Canton of Vaud (846′300 inhabitants in 2023; Etat de Vaud, 2024), the Vaud Law of Application of the Federal Law on the Adult and Child Protection states that when there are grounds for involuntary hospitalisation, but care can be provided on an outpatient basis, a CTO can be ordered (Grand Conseil du Canton de Vaud, 2012). The criteria for applying one of these coercive measures are to suffer from a mental disorder, a mental disability or a serious neglect and the fact that the required treatment or care cannot be provided otherwise. Patients’ capacity to consent is not taken into account. If the patient does not comply with the CTO, an involuntary hospitalisation can be decided by the guardianship authority, called Adult Protection Authority (APA).

Between 2013 and 2020, the 9 cantonal APA districts and eight psychiatrists authorised by the Public Health Service (PHS) could issue CTOs. Few cases were referred to authorised psychiatrists, likely due to the more demanding application process, which required a detailed justification, treatment goals, and a proposed plan—unlike APA referrals, which only required a letter. Furthermore, authorised psychiatrists had limited availability due to ongoing clinical responsibilities. Consequently, from 2021 onward, the authority to issue CTOs was assigned exclusively to civil judges of the guardianship authority (Département de la santé et de l'action sociale, 2014). The patients and their relatives can appeal against the CTO decision within ten days and request that it be lifted at any time. No forced medication is allowed under the CTOs legislation in Switzerland. The legislation does not detail the content or duration of the CTO, which is left at the discretion of the authorised psychiatrist or guardianship authority pronouncing it. However, re-evaluations are requested after 6 months, 1 year and then every year.

When the CTOs were introduced, the Canton of Vaud enshrined their monitoring in the cantonal law. In a previous article, the epidemiology of CTOs during the first 5 years following their introduction, as well as the factors associated with their duration and success were examined (Silva et al., 2019). To date, this is the first and only study focusing on CTOs’ implementation in Switzerland. It showed that CTOs prevalence increased rapidly despite the lack of evidence on positive outcomes. The results also suggested that once under CTO, it could take a long time for a patient to be released, in case of both positive and negative outcomes.

The aim of this new study was to provide up-to-date data on the use of CTOs in Western Switzerland 10 years after their introduction, to examine the profile of people undergoing CTOs during this period, the form that these measures took and the factors associated with their duration and outcome.

## Method

### Study Design

Since 2013, all CTO decisions issued by an authorised doctor or a civil judge in the Canton of Vaud have been transmitted to the PHS and data extracted through a standardised coded form. This retrospective epidemiological study focused on the analysis of the PHS’s data on CTOs pronounced between the 1st January 2013 and the 31st December 2022. Available data included sociodemographic characteristics, clinical characteristics and CTOs’ characteristics. Besides age, gender, marital status, origin (country of birth or country of descent for children born in Switzerland to non-Swiss parents) and housing conditions, information about an undergoing legal guardianship and/or involuntary hospitalisation when the CTO was ordered were also collected. PHS data provided patients’ main diagnosis, based on the ICD 10 classification, as well as information on the presence of a secondary diagnosis of addiction and/or personality disorder and of an alcohol and/or substance use secondary problem (in absence of a primary or secondary diagnosis of addiction). The existence of a danger for themselves and/or for others was also registered. Data on CTOs included who requested the measure, who pronounced it (authorised psychiatrists were only able to issue CTOs until 2020) and who was in charge for its implementation and follow up. We also knew based on which legal criteria the CTO was pronounced and what was its content. Finally, CTO status at the 31st December 2022 (underway or discharged), the date of CTO order and eventual discharge, and the reasons for discharge (CTO success; breached conditions without involuntary hospitalisation; breached conditions with involuntary hospitalisation; death; other) were also collected.

All data were anonymized and the study approved by the Swiss Ethics Committee on research involving human of Lausanne (N. 2022–00688).

### Statistical Analysis

The incidence rate of CTOs corresponded to the number of new measures pronounced each year between 2013 and 2022 per 100′000 inhabitants. The point prevalence rate of CTOs corresponded to the total number of measures underway on the 31st December of each year between 2013 and 2022 per 100′000 inhabitants.

Descriptive analyses were carried out on sociodemographic and clinical characteristics, as well as on CTOs’ characteristics. Kaplan–Meier survival analysis was used to estimate time to discharge on the whole sample (N = 530). The time point of CTO order was introduced as time origin. The time point of CTO discharge or the date of the end of data collection for ongoing CTOs (fixed at the 31st December 2022) was introduced as end-point.

Factors associated with shorter time to discharge were estimated through a series of Cox’s proportional hazards regression models. CTO’s status was used as dependent variable (event = discharge) and number of days under CTO as time variable. Sociodemographic, clinical and CTO’s characteristics were first tested in univariate models. All variables reaching a p < 0.10 level of significance in the univariate models were then introduced as one block into a multivariate final model.

Finally, to identify factors associated with CTOs’ reasons for discharge, we decided to use a Bayesian model comparison approach. This provides an attractive solution to the classic problem of multiple comparisons and allows assessing the support for the null hypothesis (Golay et al., 2019; Noël, 2015). All 52 possible models were estimated. The homogeneous model (1,2,3,4,5), which corresponds to the null hypothesis in classical statistical framework, stated that the five groups did not differ and were drawn from the same distribution. The heterogeneous model (1) (2) (3) (4) (5), on the contrary, indicated that all the groups were different and issued from different distributions. All other possible combinations up to 52 – for instance (1, 2) (3,4,5) or (1,5) (2,3,4) – were also estimated. For continuous variables, the best possible Gaussian model (μ, σ2) was identified using the Bayes information criterion (BIC; Schwarz, 1978). For nominal variables, the best multinomial model was identified using the exact likelihood with a uniform prior on all parameters (Noël, 2015). An equal prior probability of 1/52 was assumed for all models, meaning that no model was favoured. We also computed the Bayes factor (Kass & Raftery, 1995) to provide a comparison between the best model and the homogeneous model. A Bayes factor of 4 indicates that the best model was 4 times more likely to be true than the homogeneous model. Values greater than 3 are generally considered sufficiently important to favour one model over another (Jeffreys, 1961; Wagenmakers et al., 2011).

All statistical analyses were performed using IBM SPSS version 29 and the Bayes R2STATS group models calculator (Noël, 2018).

## Results

### CTOs’ Incidence and Prevalence Rates

**Fig. 1:**
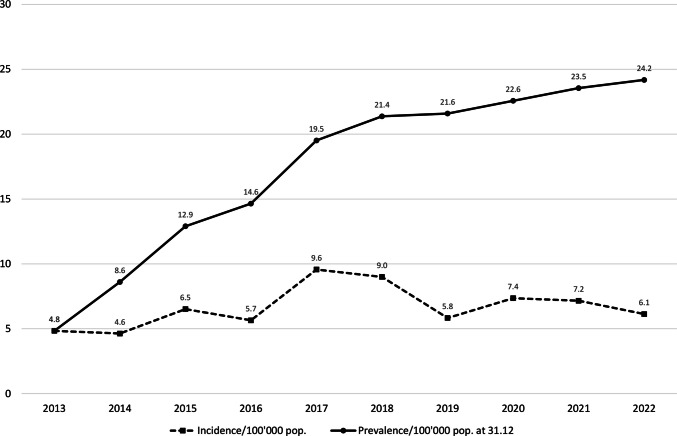
Incidence and prevalence rates of CTOs between 2013 and 2022 in the Canton of Vaud, Switzerland

Between the 1st January 2013 and the 31st December 2022, 530 CTOs have been ordered in the Canton of Vaud (504 (95.1%) by a civil judge and 26 (4.9%) by an authorised doctor. Incidence rates per 100′000 inhabitants were quite constant, fluctuating between 4.8 in 2013 and 6.1 in 2022, with a peak of 9.6 in 2017. On the contrary, CTOs’ point prevalence per 100′000 inhabitants increased between 2013 and 2022, going from 4.8 to 24.2. The annual incidence and prevalence rates of CTOs are detailed in Fig. 1.

### Sociodemographic and Clinical Characteristics of People under CTO

Socio-demographic and clinical characteristics of people under CTO between 2013 and 2022 in the Canton of Vaud, Switzerland, are detailed in Table 1. Globally, the 10 years follow up data confirmed the sociodemographic and clinical profile emerged in the previous five years study (Silva et al., 2019). People under CTO were mainly single (50.1%) or divorced/separated (31.6%) male (53.4%), aged around 49 years, living independently (71.1%) and of Swiss origin (75.5%). 69.8% of the CTO population was under legal guardianship when the CTO was pronounced. Almost half of them presented a danger for themselves (45.8%) while almost one fourth presented a danger for others (22%). The main diagnosis was confirmed to be schizophrenia, schizotypal and delusional disorders (ICD-10 F20-F29; 41%), followed by mental and behavioural disorders due to use of alcohol (ICD-10 F10; 26.6%). Data also supported the previous findings on the relevance of alcohol and/or substance use problems in this population, present not only as main diagnosis but also as comorbidity (17%) or secondary problem (9.3%).

**Table 1:** Socio-demographic and clinical characteristics of people under CTO between 2013 and 2022 in the Canton of Vaud, Switzerland (N = 530)

Characteristics	
**Age**	
Mean (SD)	49.1 (16.7)
Mdn (IQR)	49.0 (24.0)
Min/Max	15/93
**Sex, % (n)**	
Male	53.4 (283)
**Origin, % (n)**	
Swiss	75.5 (379)
**Marital status, % (n)**	
Single	50.1 (263)
Married/Registered partnership	13.1 (69)
Divorced/Separated	31.6 (166)
Widowed	5.1 (27)
**Housing conditions, % (n)**	
Independent housing	71.1 (372)
Residential centre	23.7 (124)
Homeless	4.2 (22)
Other	1.0 (5)
**Legal guardianship underway at CTO order, % (n)**	69.8 (370)
**Involuntary admission underway at CTO order, % (n)**	59.0 (311)
**Main diagnosis (ICD-10), % (n)**	
Organic, including symptomatic, mental disorders (F00-F09)	5.6 (29)
Mental and behavioural disorders due to use of alcohol (F10)	26.6 (138)
Mental and behavioural disorders due to psychoactive substance use (F11-F19)	5.4 (28)
Schizophrenia, schizotypal and delusional disorders (F20-F29)	41.0 (213)
Mood [affective] disorders (F30-F39)	7.3 (38)
Disorders of adult personality and behaviour(F60-F69)	8.3 (43)
Other	5.8 (30)
**Comorbidity (ICD-10), % (n)**	
F10-F19	17.0 (88)
F60-F69	13.5 (70)
**Alcohol and/or substance use secondary problem** (in absence of a primary or secondary diagnosis of addiction)**, % (n)**	9.3 (49)
**Danger for themselves, % (n)**	45.8 (242)
**Danger for others, % (n)**	22.0 (116)

SD Standard Deviation Mdn Median, IQR Interquartile Range

The only difference registered concerned the percentage of CTOs ordered at discharge from an involuntary admission. At 10 years follow-up a slightly lower percentage of CTOs were indeed pronounced following an involuntary admission compared to what was observed during the first five years of their implementation (59% in the 10 years follow-up study vs 67.9% in the first study).

### CTOs’ Characteristics

Characteristics of CTOs ordered between 2013 and 2022, are presented in Table 2. During the first 10 years after their introduction, most of CTOs were requested by a psychiatrist (73.8%), namely forensic, ambulatory and hospital psychiatrist. However, 504 CTOs out of 530 (95.1%) were ordered by a civil judge. A psychiatrist was also the person most frequently in charge of the CTO follow-up (81.4%). No differences emerged compared to the 2013–2017 data in terms of legal criteria justifying the measure or its content. CTOs were mainly ordered because of need of treatment (59.4%) and most patients were required to respect their appointments with a mental health professional (81.3%), allow the latter to visit them at home (50.7%) and take their medication (49.5%). Other restrictions, such as the obligation to take part in occupational activities and to accept support in daily activities were also frequently included in the CTO order (37.6%).

**Table 2:** Characteristics of CTOs ordered between 2013 and 2022 in the Canton of Vaud, Switzerland (N = 530)

Characteristics	
**CTO requested by, % (n)**	
Civil judge	10.9 (58)
Psychiatrist	73.8 (391)
General practitioner	3.0 (16)
Other	12.3 (65)
**CTO ordered by, % (n)**	
Guardianship authority	95.1 (504)
Authorised psychiatrist	4.9 (26)
**Legal criteria, % (n)**	
Treatment criterion	59.4 (315)
Dangerousness criterion	3.8 (20)
Both	35.7 (189)
Not specified	1.1 (6)
**CTO content, % (n)**	
Medication	49.5 (262)
Appointments with mental health professionals	81.3 (430)
Home visits	50.7 (268)
Addiction treatment	20.2 (107)
Blood test	18.5 (98)
Somatic treatment	31.0 (164)
Other	37.6 (199)
**Person in charge of CTO, % (n)**	
Psychiatrist	81.4 (429)
General practitioner	17.3 (91)
Non-medical professional	1.3 (7)
**CTO’s status at 31 st December 2022, % (n)**	
Underway	37.9 (201)
Discharged	62.1 (329)
**Discharge reasons, % (n)**	
CTO success	38.6 (127)
Breached conditions without involuntary hospitalisation	11.6 (38)
Breached conditions with involuntary hospitalisation	24.0 (79)
Death	13.1 (43)
Other	12.8 (42)

SD Standard Deviation Mdn Median, IQR Interquartile Range

### Factors Associated with Shorter Time to CTO Discharge

**Fig. 2:**
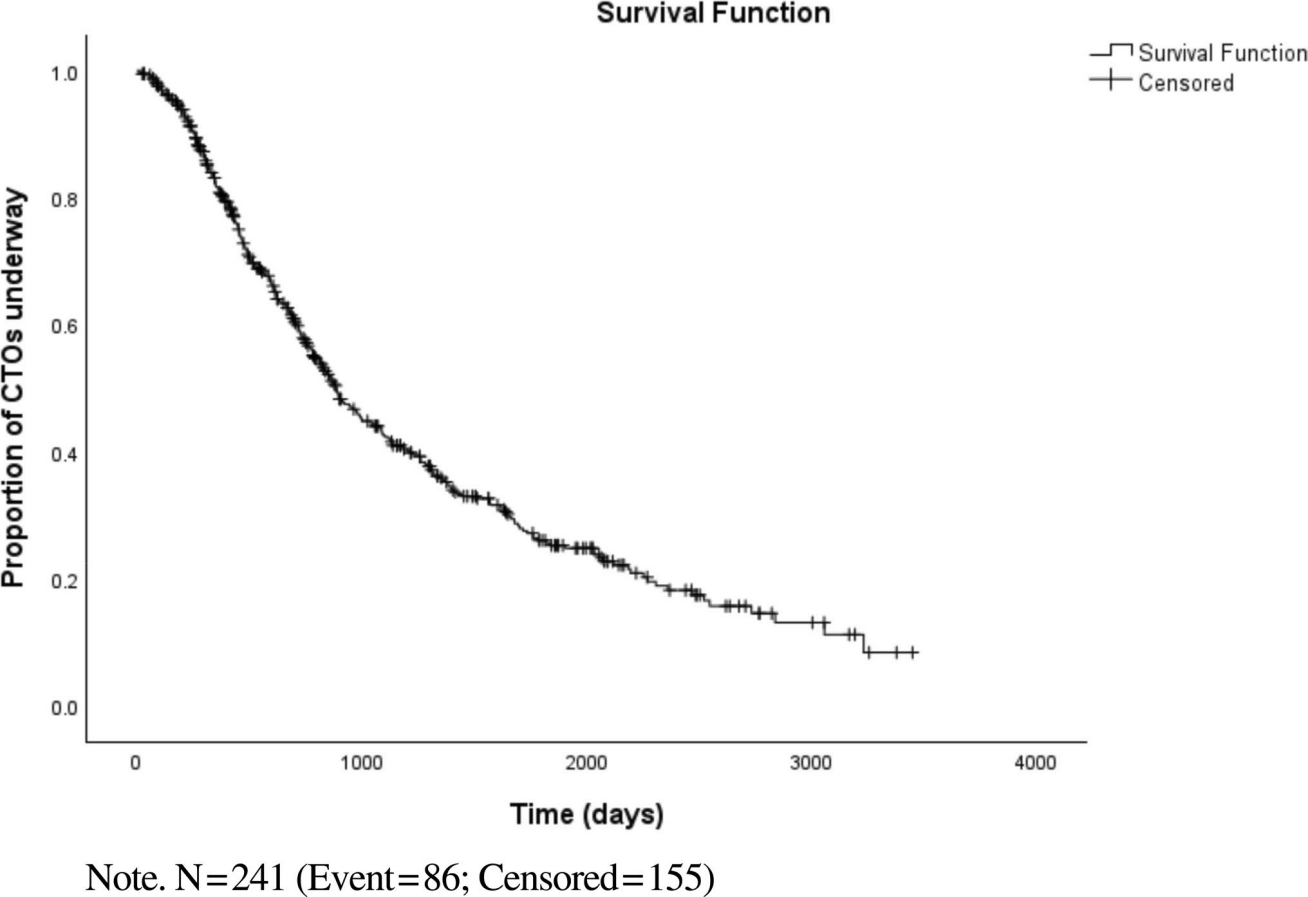
Kaplan–Meier survival curve for time to CTO discharge

On the 31st of December 2022, 329 CTOs (62.1%) were discharged while 201 (37.9%) were still underway (Table 2). Discharged CTOs lasted on average 763.2 days (SD = 590.9), with a median of 608 days (IQR = 660.0). The shortest CTO lasted 7 days and the longest 3,232 days, i.e. almost 9 years. When CTOs still underway were also taken into account in the analysis using Kaplan–Meier survival curve (N = 504), results showed that there was a 10% of chances for a CTO to last more than 8 years (Fig. 2). The resulting estimated survival mean (1286.9 days; SE = 55.5) and estimated survival median (889.0 days; SE = 50.4) were both considerably higher compared to when only discharged CTOs were analysed.

Univariate Cox’s proportional hazards regression models revealed that several factors were associated with shorter time to CTO discharge at a p < 0.10 level of significance. These factors were age, marital status, legal guardianship, danger for themselves, legal criteria, CTO requirement to take medication and follow an addiction treatment, and person in charge of the CTO (see Table 1s and 2s in Supplementary File). However, once introduced into the multivariate model, only the requirement to take medication was found to significantly increase the risk of having a longer CTO (OR = 0.733; p = 0.010; Table 3).

**Table 3:** Factors associated with shorter time to CTO discharge between 2013 and 2022 in the Canton of Vaud, Switzerland: multivariate model

Predicting factors	B (S.E.)	OR	95% C.I		p-value
**Age**	0.004 (0.004)	1.004	0.995	1.013	.369
**Marital status** (ref. Single)					
Married/Registered partnership	0.184 (0.185)	1.202	0.837	1.728	.319
Divorced/Separated	0.122 (0.145)	1.129	0.849	1.502	.403
Widowed	0.033 (0.266)	1.033	0.613	1.741	.902
**Legal guardianship underway at CTO order** (ref. No)	−0.150 (0.124)	0.861	0.675	1.097	.226
**Danger for themselves** (ref. No)	0.243 (0.137)	1.275	0.976	1.666	.075
**Legal criteria** (ref. Treatment criterion)					
Dangerousness criterion	−0.129 (0.296)	0.879	0.492	1.569	.662
Both	0.10 (0.141)	1.010	0.767	1.331	.943
Not specified	−0.444 (0.738)	0.642	0.151	2.725	.548
**CTO content** (ref. No medication)					
Medication	−0.311 (0.120)	0.733	0.579	0.928	**.010**
Addiction treatment	0.024 (0.149)	1.024	0.765	1.371	.874
**Person in charge of CTO** (ref. Psychiatrist)					
General practitioner	0.110 (0.159)	1.117	0.818	1.524	.486
Non-medical professional	0.354 (0.586)	1.425	0.452	4.490	.546

C.I = Confidence Interval; OR = Odds Ratio

χ^2^(13) = 25.197; p < 0.05; N = 520 (Event = 321; Censored = 199)

### Comparison between Groups of People under CTO According to the Reason for Discharge

Among the 329 CTOs discharged at the 31st of December 2022, five groups were identified according to the reasons for discharge (Table 2). The first group (38.6%) included all CTO cases discharged because the objectives of the measure were successfully achieved. Groups 2 (11.5%) and 3 (24.0%) concerned the CTOs withdrawn because of breach of conditions, without or with subsequent involuntary hospitalisation. Group 4 (13.0%) included all CTOs discharged following the person's death. Finally, the fifth group (12.8%) referred to ‘other’ reasons for discharge, such as the voluntary admission to a residential centre or the implementation of a penal measures.

When the five groups were compared on their sociodemographic and clinical characteristics, several differences could be identified (Table 4). First, we observed that patients in group 4 (Death) were significantly older than those in the other groups (mean age = 58.8 years; SD = 15.4) and more frequently separated or divorced (52.4%). Fewer people were under legal guardianship in group 1 (CTO success; 57.5%) and group 4 (Death; 55.8%). Patients in group 5 (Other) were more likely to present a main diagnosis of organic mental disorders (F00-F09) and ‘other’ diagnoses. Finally, more people presented a danger for themselves in group 3 (Breached conditions with IH; 58.2%) and group 4 (Death; 67.4%).

**Table 4:** Comparison of sociodemographic and clinical characteristics between groups of patients presenting different discharge reasons (N = 329)

	(1) CTO success N = 127	(2) Breached conditions without IH N = 38	(3) Breached conditions with IH N = 79	(4) Death N = 43	(5) Other N = 42	Best model^a^	Bayes factor against null hypothesis^b^	Probability of the model to be true
**Age**, mean (SD)	48.5 (16.4)	43.8 (17.1)	50.6 (15.4)	58.8 (15.4)	50.5 (22.1)	(1, 2, 3, 5) (4)	33.2286	0.4099
**Sex, % (n)**								
Male	47.2 (60)	63.2 (24)	54.4 (43)	53.5 (23)	54.8 (23)	(1, 2, 3, 4, 5)	1.0000	0.1322
**Origin** % (n)								
Swiss	75.6 (93)	66.7 (24)	78.9 (60)	75.6 (31)	70.7 (29)	(1, 2, 3, 4, 5)	1.0000	0.1905
**Marital status, % (n)**								
Single	45.2 (57)	58.3 (21)	46.8 (37)	21.4 (9)	46.3 (19)	(1, 2, 3, 5) (4)	6.2059	0.2849
Married/Registered partnership	17.5 (22)	5.6 (2)	12.7 (10)	16.7 (7)	26.8 (11)			
Divorced/Separated	31.0 (39)	33.3 (12)	38.0 (30)	52.4 (22)	17.1 (7)			
Widowed	6.3 (8)	2.8 (1)	2.5 (2)	9.5 (4)	9.8 (4)			
**Housing conditions, % (n)**								
Independent housing	76.0 (95)	73.0 (27)	70.9 (56)	67.4 (29)	76.2 (32)	(1, 2, 3, 4, 5)	1.0000	0.8564
Residential centre	19.2 (24)	13.5 (5)	26.6 (21)	25.6 (11)	16.7 (7)			
Homeless	4.0 (5)	10.8 (4)	2.5 (2)	7.0 (3)	4.8 (2)			
Other	0.8 (1)	2.7 (1)	0.0 (0)	0.0 (0)	2.4 (1)			
**Legal guardianship underway at CTO order, % (n)**	57.5 (73)	68.4 (26)	75.9 (60)	55.8 (24)	76.2 (32)	(1, 4) (2, 3, 5)	27.5629	0.2507
**Involuntary admission underway at CTO order, % (n)**	62.1 (77)	50.0 (19)	64.6 (51)	60.5 (26)	61.9 (26)	(1, 2, 3, 4, 5)	1.0000	0.1699
**Main diagnosis (ICD-10), % (n)**								
F00-F09	3.2 (4)	5.3 (2)	2.6 (2)	9.5 (4)	23.8 (10)	(1, 2, 3, 4) (5)	103.2341	0.6833
F10	29.0 (36)	21.1 (8)	37.2 (29)	38.1 (16)	16.7 (7)			
F11-F19	4.8 (6)	13.2 (5)	2.6 (2)	9.5 (4)	0.0 (0)			
F20-F29	37.9 (47)	36.8 (14)	43.6 (34)	26.2 (11)	38.1 (16)			
F30-F39	12.9 (16)	2.6 (1)	5.1 (4)	9.5 (4)	4.8 (2)			
F60-F69	5.6 (7)	15.8 (6)	6.4 (5)	4.8 (2)	4.8 (2)			
Other	6.5 (8)	5.3 (2)	2.6 (2)	2.4 (1)	11.9 (5)			
**Comorbidity (ICD-10), % (n)**								
F10	13.7 (17)	10.5 (4)	15.4 (12)	16.7 (7)	11.9 (5)	(1, 2, 3, 4, 5)	1.0000	0.3005
F60-F69	12.9 (16)	15.8 (6)	15.4 (12)	23.8 (10)	9.5 (4)	(1, 2, 3, 4, 5)	1.0000	0.1868
**Alcohol and/or substance use secondary problem, % (n)**	5.5 (7)	13.2 (5)	11.4 (9)	7.0 (3)	14.3 (6)	(1, 2, 3, 4, 5)	1.0000	0.1736
**Danger for themselves, % (n)**	43.7 (55)	50.0 (19)	58.2 (46)	67.4 (29)	47.6 (20)	(1, 2, 5) (3, 4)	6.6560	0.1616
**Danger for others, % (n)**	23.0 (29)	28.9 (11)	16.5 (13)	25.6 (11)	28.6 (12)	(1, 2, 3, 4, 5)	1.0000	0.1614

^a^On the basis of the BIC coefficient

^b^Bayes factor comparing the best model with the homogeneous model (1,2,3,4,5)

^c^Among all 52 possible models

Group differences were also observed on CTOs characteristics (Table 5). CTOs were longer in group 1 (CTO success; mean = 898.5; SD = 652.8) and group 4 (Death; mean = 778.2; SD = 630.0). Regarding the content of the CTOs, home visits were rarely planned in group 2 (Breached conditions without IH; 22.6%) but very frequently used in group 3 (Breached conditions with IH; 63.3%) and group 4 (Death; 72.1%). Addiction treatment was less frequent in group 1 (CTO success; 15.7%) and group 5 (Other; 11.9%) while somatic treatment was more frequently required in group 4 (Death; 58.1%).

**Table 5:** Comparison of CTOs’ characteristics between groups of patients presenting different discharge reasons (N = 329)

	(1) CTO success N = 127	(2) Breached conditions without IH N = 38	(3) Breached conditions with IH N = 79	(4) Death N = 43	(5) Other N = 42	Best model^a^	Bayes factor against null hypothesis^b^	Probability of the model to be true
**Time to discharge**, mean (SD)	898.5 (652.8)	689.9 (465.7)	600.1 (522.2)	778.2 (630.0)	711.9 (496.9)	(1, 4) (2, 3, 5)	15.6571	0.2286
**CTO requested by, % (n)**								
Civil judge	9.4 (12)	15.8 (6)	11.4 (9)	7.0 (3)	16.7 (7)	(1, 2, 3, 4, 5)	1.0000	0.8767
Psychiatrist	74.0 (94)	78.9 (30)	74.7 (59)	74.4 (32)	71.4 (30)			
General practitioner	3.9 (5)	0.0 (0)	2.5 (2)	4.7 (2)	2.4 (1)			
Other	12.6 (16)	5.3 (2)	11.4 (9)	14.0 (6)	9.5 (4)			
**Legal criteria, % (n)**								
Treatment criterion	60.6 (77)	63.2 (24)	54.4 (43)	44.2 (19)	57.1 (24)	(1, 2, 3, 4, 5)	1.0000	0.7216
Dangerousness criterion	4.7 (6)	5.3 (2)	1.3 (1)	2.3 (1)	11.9 (5)			
Both	33.9 (43)	28.9 (11)	43.0 (34)	51.2 (22)	31.0 (13)			
Not specified	0.8 (1)	2.6 (1)	1.3 (1)	2.3 (1)	0.0 (0)			
**CTO content, % (n)**								
Medication	52.0 (66)	37.8 (14)	50.6 (40)	37.2 (16)	42.9 (18)	(1, 3) (2, 4, 5)	1.3449	0.1225
Appointments with mental health professionals	84.3 (107)	91.9 (34)	81.0 (64)	76.7 (33)	78.6 (33)	(1, 2, 3, 4, 5)	1.0000	0.1782
Home visits	48.8 (62)	21.6 (8)	63.3 (50)	72.1 (31)	50.0 (21)	(1, 5) (2) (3, 4)	7691.9285	0.3802
Addiction treatment	15.7 (20)	32.4 (12)	25.3 (20)	32.6 (14)	11.9 (5)	(1, 5) (2, 3, 4)	14.0995	0.2800
Blood test	18.9 (24)	18.9 (7)	20.3 (16)	14.0 (6)	11.9 (5)	(1, 2, 3, 4, 5)	1.0000	0.2423
Somatic treatment	30.7 (39)	21.6 (8)	30.4 (24)	58.1 (25)	23.8 (10)	(1, 2, 3, 5) (4)	219.7644	0.3470
Other	38.6 (49)	21.6 (8)	40.5 (32)	44.2 (19)	45.2 (19)	(1, 3, 4, 5) (2)	2.8358	0.2197
**Person in charge of CTO, % (n)**								
Psychiatrist	81.9 (104)	94.7 (36)	80.8 (63)	74.4 (32)	66.7 (28)	(1, 2, 3, 4) (5)	1.0743	0.3179
General practitioner	18.1 (23)	5.3 (2)	17.9 (14)	25.6 (11)	28.6 (12)			
Non-medical professional	0.0 (0)	0.0 (0)	1.3 (1)	0.0 (0)	4.8 (2)			

^a^On the basis of the BIC coefficient

^b^Bayes factor comparing the best model with the homogeneous model (1,2,3,4,5)

^c^Among all 52 possible models

## Discussion

### CTOs’ Incidence and Prevalence Rates

The results of this study have shown that over the ten-years observation period, the incidence rates of CTOs in the Canton of Vaud have remained relatively low and stable. Indeed, it fluctuated between 5 and 10 CTOs per 100′000 inhabitants. Conversely, the prevalence rate rose from 5 CTOs per 100′000 inhabitants at the end of 2013 to 25 CTOs per 100′000 inhabitants at the end of 2022. The increment was particularly pronounced between 2013 and 2017, suggesting that each year more CTOs were pronounced than discharged. Since 2018, the growth in prevalence has begun to slow down indicating that a balance between the number of CTO issued and discharged may be approaching. It is important to note that no direct link was observed between the increase in the prevalence of CTOs and the change in the number of involuntary hospitalisations in the canton (Etat de Vaud, 2022). This observation is consistent with the results of previous studies (Maughan et al., 2014).

### Sociodemographic and Clinical Characteristics of People under CTO

The sociodemographic and clinical profile of people placed under CTO has remained remarkably constant over the years and is comparable to that observed in other studies. (Silva et al., 2019). The only notable change compared to the 2013–2017 study (Silva et al., 2019) was that in the 10-year observation study slightly fewer people were involuntarily hospitalised when the CTO was pronounced (59.0% vs. 67.9% at 5 years). This trend can be explained by the various actions that, from 2017, the Canton of Vaud has taken to attempt to curb the steady increase in involuntary hospitalisations rates observed between 2013 and 2016: from 2.8 involuntary hospitalisation per 1′000 inhabitants in 2013 to 3.2 in 2016 (+ 14%; Etat de Vaud, 2022). Firstly, procedures governing involuntary hospitalisations were revised in order to guarantee the respect for patients'rights. Secondly, collaboration between mental healthcare professionals and the justice system was strengthened. Thirdly, the skills of these two professional groups were improved through training courses. These actions were followed by a significant reduction of 19% in the incidence rate of involuntary hospitalisations between 2017 and 2022 (2.6 involuntary hospitalisation per 1′000 inhabitants in 2022).

### CTOs’ Characteristics

After 10 years from their introduction, the characteristics of the CTOs, origin of the request, reasons justifying it, and content of the measures, were comparable to what observed in the previous five-years follow-up epidemiological study (Silva et al., 2019). The proportion of measures discharged has risen steadily over time (62.1% compared to 35.7% after five years). To prevent these measures from become never ending conditions a closer collaboration between civil judges and mental healthcare professionals was promoted and trainings was provided to them. Consequently, civil judges, who are now the only ones allowed to pronounce CTOs, have acquired a better understanding of the therapeutic possibilities offered by these measures, as well as their limitations, making it easier for them to revoke a CTO. Despite that, the average duration of CTOs was slightly longer (1286.9 days after 10 years compared to 1060.1 days after five years) and very long CTO were still present.

### Factors Associated with Shorter Time to CTO Discharge

Multivariate regression analysis showed that only the requirement to take a medication was correlated with longer time spent on CTO. Thus, conversely to what observed in the previous five-year epidemiological study, not being Swiss was no longer associated with a longer CTO, as well as living condition and the professional background of the person in charge of the CTO were no longer associated with a shorter time to discharge. A first hypothesis that could explain the link between the obligation to take a medication and the prolonged duration of CTO could be that these patients being cooperative and having a favourable outcome under CTO, the professionals may have been reluctant to revoke the measure. A second hypothesis may on the contrary concern situations of patients with severe mental disorders. The Swiss legal framework does not provide for forced medication as part of a CTO (The Federal Authorities of the Swiss Confederation, 2024). However, when it comes to involuntary hospitalisations, it is mentioned that a treatment without consent may be prescribed if a failure to carry out the treatment could lead to serious damage to the patient's health or seriously endanger the life or the physical integrity of third parties; the patient is unable to exercise judgement in relation to his or her need for treatment; and no appropriate measure is available that is less invasive. Judges and doctors may thus have considered forced medication as part of a CTO for situations that meet these same criteria and consequently be more reluctant to lift the measure with these patients.

### Comparison Between Groups of People under CTO According to the Reason for Discharge

Of the 329 CTOs lifted at the end of the 10-year observation period, 38.7% had been lifted because of the patient's positive outcome. 35.5% of the measures had been revoked because of breach of conditions, 24% followed by an involuntary hospitalisation and 11.5% without it, and 13% for other reasons. Finally, 12.8% of the patient under CTOs had died. By examining the characteristics of patients and of CTOs at the time of the order in the light of the reason for discharge, a prototypical profile of the situations that make up these different groups can be drawn.

#### CTO Success: “Less Complex Patients”

In the group where the CTO was revoked due to the success of the measure, patients were less regularly under legal guardianship. This could be explained by the fact that psychosocial difficulties were less significant in this group than in the others. These situations could therefore be considered less complex, all the more so, as addiction treatment was less often offered in this group. Finally, the CTO lasted longer in this group than in the others. This could confirm that when professionals feel that the measure is working, they are reluctant to revoke it (Riley et al., 2018). This difficulty in revoking the CTO independently of the evolution of the situation has often been referred to as the “lobster pot” effect (Maylea et al., 2025). The way in which professionals manage the risk of a possible relapse also plays a decisive role in decisions regarding the continuation of a CTO. Previous studies have indicated that CTOs were sometimes maintained due to the absence of alternative care options. While access to outpatient mental health care in Switzerland is the same for people with and without CTOs, models such as assertive community treatment are scarce (Morandi, 2018). In this context, coercion may therefore be seen as the only way to engage patients in care. The association between CTO success and prolonged duration of CTO could also confirm the results of previous studies, which showed that outcomes were more favourable for CTOs lasting longer than two years (Kisely et al., 2024).

#### Breached Conditions without Involuntary Hospitalisation: “Less Accessible, More Refractory Patients”

In comparison with the other groups, it is interesting to note that among the situations where the CTO conditions were breached, but without the need for an involuntary hospitalisation, the initial CTO decision required less regularly home visits and other socio-educational measures. The more limited number of therapeutic measures implemented could be interpreted as due to the professionals not being totally convinced of the usefulness of coercion in these situations or believing that patients would not comply with or benefit from additional measures. Another explanation could be that the health status of some patients in this group improved despite the lack of collaboration with the healthcare system. A previous study had already shown that some patients who were difficult to access or refractory did not require psychiatric hospitalisation, despite the absence of contact with outpatient care (Golay et al., 2022).

#### Breached Conditions with Involuntary Hospitalisation: “Severely Ill Patients Needing Protection”

Compared with the other groups (with the exception of the ‘death’ group), a danger for themselves was more regularly mentioned in the CTO decision of patients whose measure was finally revoked and replaced by an involuntary hospitalisation. They were very frequently under legal guardianship at CTO order. The CTO decision also made more regular provision for home visits and addiction treatment. It can thus be assumed that this group concerned complex clinical situations for which numerous coercive ambulatory therapeutic measures were proposed, in order to stabilise the patient's mental health. However, these proved insufficient and ultimately necessitated an involuntary hospitalisation.

#### Death: “Patients with Life Threatening Conditions”

Patients in the “death” group were older. They were more often separated or divorced, but less often under legal guardianship. This last point could be explained by the fact that in this group the need for protection primarily concerned health, while the patients’ management of administrative affairs was not necessarily a problem. The CTO decision more often mentioned a danger for themselves. Time to CTO discharge was longer. Patients were more often required to undergo home visits, addiction treatment and, much more frequently than in the other groups, somatic treatment. We can therefore hypothesise that these patients had somatic comorbidities for which they required treatment, some of them possibly related to their substance abuses. The older age of the patients, although only around 59 on average, substance abuse and somatic pathologies may explain the higher mortality in this group.

#### Other Reason for Discharge: “Patients with Neurodegenerative Disorders”

In this group, patients suffered more regularly from organic disorders and not surprisingly were more frequently under legal guardianship. The doctor in charge of the CTO was more often a non-psychiatrist than in the other groups. The worsening of the neurodegenerative disorders may have led to the need to place the patients concerned in specialised institutions, without them opposing this decision.

## Conclusions

Since the introduction of CTOs, their use has remained limited in the Canton of Vaud. Although the legal framework does not set a maximum duration, discharged CTOs lasted just over two years. The profile of patients placed under CTO remained remarkably stable over the ten years of observation. When we look at the associations between the CTO outcome, patients' characteristics and the content of the CTO decision, we identify groups with specific profiles according to the outcome of the measure.

Although our study showed that some patients improved while under CTO, it is not possible to establish the role that coercion played in these situations. Furthermore, in over a third of cases, the CTO was revoked because its conditions were not respected. We can thus conclude that CTOs do not enhance the commitment to care of certain patients who are refractory or not accessible to care. Furthermore, these measures do not replace institutional placements, which remain necessary in certain complex situations. Finally, the fact that more than one in ten patients placed under CTO died at a relatively young age during the observation period is a reminder that psychiatric disorders are often associated with serious somatic problems. It is therefore essential to continue to study the use of coercion, in particular among populations with specific needs and to search alternatives whenever possible.

## Supplementary Information

Below is the link to the electronic supplementary material.

Supplementary file1 (DOCX 22 KB)
