# Supplementary material for: Ten Years of Community Treatment Orders in Western Switzerland: an Update
Source: Community Ment Health J. 2025 Jul 18;61(8):1513–23. doi: 10.1007/s10597-025-01486-5 (PMC12647211; doi:10.1007/s10597-025-01486-5)
Supplement: Supplementary file 1 — Supplementary file1 (DOCX 22 KB) [file 10597_2025_1486_MOESM1_ESM.docx]

**Supplementary File :**

| **Table 1s. Sociodemographic and clinical characteristics associated with shorter time to CTO discharge between 2013 and 2022 in the Canton of Vaud, Switzerland: univariate model** | | | | | | | | |  |
| --- | --- | --- | --- | --- | --- | --- | --- | --- | --- |
| **Predicting factors** | **B (S.E.)** | **OR** | | **95% C.I.** | | | **p-value** | |  |
| **Age** | 0.008 (0.004) | | 1.008 | | 1.001 | 1.015 | | **.021** | |
|  |  | |  | |  |  | |  | |
| **Sex** (ref. Male) | | | | | | | | |  |
| Female | 0.74 (0.111) | | 1.077 | | 0.867 | 1.338 | | .501 | |
|  |  | |  | |  |  | |  | |
| **Origin** (ref. Swiss) | | | | | | | | |  |
| Other | -0.113 (0.130) | | 0.893 | | 0.693 | 1.152 | | .384 | |
|  |  | |  | |  |  | |  | |
| **Marital status** (ref. Single) | | | | | | | | |  |
| Married/Registered partnership | 0.321 (0.162) | | 1.378 | | 1.003 | 1.894 | | **.048** | |
| Divorced/Separated | 0.245 (0.127) | | 1.278 | | 0.996 | 1.639 | | **.054** | |
| Widowed | 0.205 (0.244) | | 1.228 | | 0.760 | 1.982 | | .402 | |
|  |  | |  | |  |  | |  | |
| **Housing conditions** (ref. Independent housing) | | | | | | | | |  |
| Residential centre | -0.180 (0.138) | | 0.835 | | 0.637 | 1.093 | | .190 | |
| Homeless | -0.289 (0.259) | | 0.749 | | 0.451 | 1.245 | | .265 | |
| Other | 0.170 (0.582) | | 1.185 | | 0.379 | 3.706 | | .770 | |
|  |  | |  | |  |  | |  | |
| **Legal guardianship underway at CTO order** (ref. No**)** | -0.228 (0.116) | | 0.796 | | 0.634 | 0.999 | | **.049** | |
|  |  | |  | |  |  | |  | |
| **Involuntary admission underway at CTO order** (ref. No) | -0.025 (0.114) | | 0.975 | | 0.781 | 1.219 | | .827 | |
|  |  | |  | |  |  | |  | |
| **Main diagnosis** (ref. F00-F09) | | | | | | | | |  |
| F10 | 0.124 (0.237) | | 1.132 | | 0.711 | 1.801 | | .603 | |
| F11-19 | -0.444 (0.324) | | 0.641 | | 0.340 | 1.211 | | .171 | |
| F20-F29 | -0.291 (0.232) | | 0.748 | | 0.475 | 1.178 | | .210 | |
| F30-39 | -0.207 (0.288) | | 0.813 | | 0.462 | 1.430 | | .473 | |
| F60-F69 | -0.277 (0.302) | | 0.758 | | 0.420 | 1.371 | | .360 | |
| Other | -0.242 (0.318) | | 0.785 | | 0.421 | 1.465 | | .447 | |
|  |  | |  | |  |  | |  | |
| **Comorbidity** (ref. No) | | | | | | | | |  |
| F10 | -0.219 (0.161) | | 0.804 | | 0.586 | 1.102 | | .175 | |
| F60-F69 | -0.013 (0.157) | | 0.987 | | 0.726 | 1.343 | | .934 | |
|  |  | |  | |  |  | |  | |
| **Alcohol and/or substance use secondary problem** (ref. No) | -0.145 (0.192) | | 0.865 | | 0.593 | 1.261 | | .451 | |
|  |  | |  | |  |  | |  | |
| **Danger for themselves** (ref. No) | 0.295 (0.111) | | 1.343 | | 1.081 | 1.668 | | **.008** | |
|  |  | |  | |  |  | |  | |
| **Danger for others** (ref. No) | -0.045 (0.131) | | 0.956 | | 0.739 | 1.235 | | .729 | |
|  |  | |  | |  |  | |  | |
| Note. C.I = Confidence Interval; OR = Odds Ratio. | | | | | | | | |  |

| **Table 2s. CTOs characteristics associated with shorter time to CTO discharge between 2013 and 2022 in the Canton of Vaud, Switzerland: univariate model** | | | | | | |
| --- | --- | --- | --- | --- | --- | --- |
| **Predicting factors** | | **B (S.E.)** | **OR** | **95% C.I.** | | **p-value** |
| **CTO requested by** (ref. Civil judge) | | | | | | |
| Psychiatrist | 0.058 (0.177) | | 1.059 | 0.749 | 1.498 | .744 |
| General practitioner | -0.227 (0.357) | | 0.797 | 0.396 | 1.604 | .525 |
| Other | 0.129 (0.233) | | 1.137 | 0.720 | 1.796 | .581 |
|  |  | |  |  |  |  |
| **Legal criteria** (ref. Treatment criterion) | | | | | | |
| Dangerousness criterion | 0.304 (0.270) | | 1.355 | 0.799 | 2.298 | .260 |
| Both | 0.225 (0.116) | | 1.253 | 0.998 | 1.574 | **.053** |
| Not specified | 0.596 (0.506) | | 1.815 | 0.673 | 4.894 | .239 |
|  |  | |  |  |  |  |
| **CTO content** (ref. No) | | | | | | |
| Medication | -0.367 (0.111) | | 0.693 | 0.557 | 0.861 | **<.001** |
| Appointments with mental health professionals | -0.105 (0.146) | | 0.900 | 0.676 | 1.198 | .471 |
| Home visits | 0.090 (0.111) | | 1.094 | 0.881 | 1.359 | .416 |
| Addiction treatment | 0.225 (0.134) | | 1.252 | 0.962 | 1.630 | **.094** |
| Blood test | -0.044 (0.145) | | 0.957 | 0.720 | 1.271 | .760 |
| Somatic treatment | 0.153 (0.118) | | 1.166 | 0.924 | 1.470 | .195 |
| Other | 0.111 (0.114) | | 1.117 | 0.894 | 1.396 | .329 |
|  |  | |  |  |  |  |
| **Person in charge of CTO** (ref. Psychiatrist) | | | | | | |
| General practitioner | 0.264 (0.142) | | 1.302 | 0.987 | 1.719 | **.062** |
| Non-medical professional | 0.213 (0.581) | | 1.238 | 0.396 | 3.867 | .714 |

Note. C.I = Confidence Interval; OR = Odds Ratio.
